# An increasing citation black hole in ecology and evolution

**DOI:** 10.1002/ece3.1356

**Published:** 2014-12-17

**Authors:** Anthony R Rafferty, Bob B M Wong, David G Chapple

**Affiliations:** School of Biological Sciences, Monash UniversityClayton, Victoria, 3800, Australia

**Keywords:** *h*-Index, journal impact factor, online supplementary material

## Abstract

Citations published in online supplementary material (OSM) are invisible to search engines used to calculate citation counts, potentially negatively impacting popular performance indices and journal rankings that rely on citation counts for quantification. To quantify the number of citations that are “lost” in OSM, we conducted a systematic survey of supplementary citation practices in four high-ranking, society-run journals from two geographical locations (Europe and North America). In 2012, 6% of all citations were only included in the OSM and were therefore not included in citation counts. We found a significant increase in the number of references invisible to citation counting services over the last two decades. A solution to this problem is urgently required and could include journal indexing of citations in OSM or the inclusion of all references in the main text.

## Measuring Success

Publication records and citation counts are commonly used to quantify the impact and significance of an individual's, department's, or institution's research. Both variables act as a proxy for academic performance and are used to calculate popular output measures including the *h*-index (Hirsch 2005). Such indices are heavily relied upon to inform decisions regarding faculty recruitment, promotion, tenure, and funding (Garfield and Welljams-Dorof 1992; Adam 2002), as well as gauge departmental and institutional performance, discipline development (Peters 1991), and scientific output on a national scale (King 2004). Citation counts are also used to calculate journal impact factors (Kurmis 2003), therefore having far-reaching implications if undercounted.

Using citation counts to evaluate academic merit is based on the assumptions that influential publications are cited more frequently (Meho 2006), and sources of information are credited appropriately. However, word restrictions imposed by many journals either result in the omission of relevant citations from the published work (MacRoberts and MacRoberts 1989) or the inclusion of such citations in online supplementary material (OSM). However, citations published in OSM are not recognized by search engines including PubMed, Scopus, Web of Science, and Google Scholar, which negatively affects citation counts (Seeber 2008). As a result, a substantial proportion of all references published in primary literature may potentially go uncounted (Weiss et al. 2010), which might significantly decrease performance indices and journal rankings.

The aim of this article was to quantify the number of citations that are “lost” in OSM, and investigate OSM citation patterns over the last two decades in four highly ranked, society-run Evolution and Ecology journals. Given the reported parochial citation practices that exist in Europe and North America (Leimu and Koricheva 2005; Wong and Kokko 2005), OSM citations in each mentioned discipline were compared between these regions.

## Quantifying the Extent of Citations in Online Supplementary Material

We conducted a systematic survey of supplementary citation practices in four journals from two geographical locations. We focused on manuscripts published in Journal of Evolutionary Biology (JEB, published in Europe; *n *=* *288), Journal of Animal Ecology (JAE, published in Europe; *n *=* *289), Evolution (published in USA; *n *=* *337), and Ecology (published in USA; *n *=* *399). Citation number was determined for manuscripts and corresponding online supplementary material (OSM, if it existed) in four issues of each journal in each of the years 1992, 2002, 2007, and 2012 (only three issues were available in 1992 in JAE). 1992 was selected as it represents a baseline prior to OSM, 2002 and 2007 were transitional periods where OSM was being increasingly adopted by journals, and 2012 represents the current state of OSM in the four journals. Manuscript type was obtained from Web of Science, as was manuscript citation number, when possible. If the latter was not provided, citations were counted manually. Only research articles, notes, concepts, comments, reports, and letters were included in this study. One meta-analysis article (Journal of Evolutionary Biology, 2002) was excluded as an outlier as well as two research articles (Journal of Animal Ecology, 2012) with broken links to OSM.

## A Developing Citation Black Hole?

**Figure 1:**
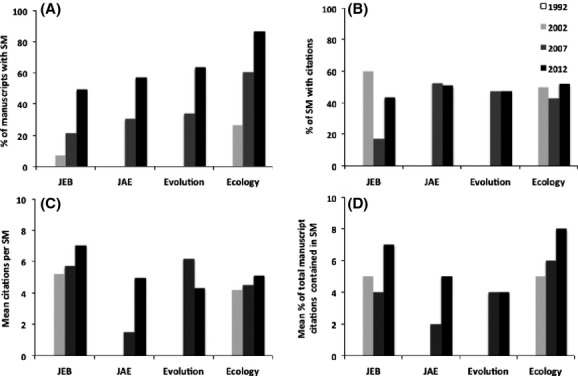
(A) Percentage of manuscripts with supplementary material (SM); (B) percentage of SM with citations; (C) mean number of citations per SM; and (D) mean percentage of SM citations relative to in-text citations. Journals surveyed include Journal of Evolutionary Biology (JEB), Journal of Animal Ecology (JAE), Evolution, and Ecology. White, light gray, dark gray, and black bars represent the years 1992, 2002, 2007, and 2012, respectively.

A total of 1313 manuscripts were surveyed for this study, 362 (28%) of which had OSM. Between 1992 and 2012, there was an increase in the percentage of manuscripts with OSM across all four journals (Fig.1A). Although no manuscripts had OSM in 1992 (or in 2002 in JAE and Evolution), an overall mean of 64% had OSM in 2012.

A similar increase over time was also observed in the percentage of OSM with citations, except for a general decrease from 2002 to 2012 in JEB (Fig.1B). Of the total 362 manuscripts with OSM surveyed during this study, 168 (46%) had 1363 citations. In 2012, the percentage of OSM with citations was relatively consistent across all four journals and exceeded 40%.

From 1992 to 2012, the mean number of citations per OSM also increased in each journal, except Evolution, which decreased between 2007 and 2012 (Fig.1C). An overall mean increase from 0 to 5 citations per OSM was observed across all four journals during the past two decades.

Finally, the number of OSM citations relative to in-text citations generally increased across all four journals between 1992 and 2012 (Fig.1D). An overall mean relative increase from 0% in 1992 to 6% in 2012 was observed across all four journals.

## Credit Where Credit is due: The Impact of Missing Citations

During the past two decades, there has been a dramatic increase in the number of manuscripts published with OSM, as well as the number of citations included in OSM. In 2012, an average 6% of all references (in-text and OSM) were published in OSM and as a result, remained invisible to services used for citation counting. These findings highlight the growing issue of citation undercounting and the implications that this may have on calculating performance indices reliant on citation counts.

For example, citation undercounting has the potential to decrease an individual's *h*-index, which is determined by the publication number and associated citation count of each article that they publish. To test for this, we examined how differences in the rate of citation undercounting could affect the *h*-index of the academic staff in our department (Table1). Although the current level of citation undercounting (4–8%) results in little change in an individuals’ *h*-index (if at all), if the increasing citation black hole continues above 10%, the change in *h*-index will become more substantial. If so, researchers risk not receiving appropriate credit for their work, which could inevitably have a detrimental effect on an individual's relative standing within a research community. This may also have a negative impact on public funding and career assessment (Kelly and Jennions 2006). Similarly, journal impact factors are used as a benchmark of journal quality and represent the mean citation rate of all manuscripts published by a particular journal (Garfield 1972). Citation undercounting therefore has the ability to decrease journal impact factors.

**Table 1:** Potential increase (mean ± SE; range) in an individuals’ *h*-index at different rates of citation undercounting. The estimate was based on the *h*-indexes and citation record (obtained from Google Scholar on the 8th November 2014) of academics in our department. Career stage follows the Australian academic system

		Rate of citation undercounting						
Academic career stage	*n*	2%	4%	6%	8%	10%	15%	20%
B (Lecturer)	6	0	0	0.2 ± 0.2 (0–1)	0.2 ± 0.2 (0–1)	0.5 ± 0.2 (0–1)	0.5 ± 0.2 (0–1)	0.8 ± 0.3 (0–2)
C (Senior Lecturer)	5	0	0	0	0	0.2 ± 0.2 (0–1)	0.6 ± 0.2 (0–1)	1.0 ± 0.0 (1–1)
D (Associate Professor)	9	0	0.1 ± 0.1 (0–1)	0.2 ± 0.1 (0–1)	0.3 ± 0.2 (0–1)	0.4 ± 0.2 (0–1)	1.0 ± 0.3 (0–2)	1.4 ± 0.3 (0–3)
E (Professor)	6	0	0.5 ± 0.2 (0–1)	1.0 ± 0.3 (0–2)	1.3 ± 0.3 (0–2)	1.5 ± 0.3 (0–2)	2.0 ± 0.4 (0–3)	2.8 ± 0.2 (2–3)

Evidently, citation undercounting has the potential to lower both *h*-indices and journal impact factors, so in theory, both academics and journals are disadvantaging themselves by publishing citations in OSM. An obvious solution to this problem would be for journals to provide indexing services for all citations, including those in OSM, or include OSM citations in the bibliography of the main document (Seeber 2008). But does this phenomenon impact everyone equally? To investigate this, we quantified and compared the mean 2012 impact factors of citations in text (*n *=* *126) and in the corresponding OSM (*n *=* *74) of five manuscripts published in Ecology in 2012. Manuscripts were randomly selected from two issues in volume 93. A Wilcoxon rank-sum test showed that the overall mean impact factor (±SD) of in-text citations (6.79 ± 6.18) was significantly higher than those included in OSM (3.86 ± 3.66; *W *=* *2812.5, *P *<* *0.001). Although limited, this investigation clearly indicates that a bias exists toward inclusion of citations from journals with a higher impact factor in text rather than in OSM. This finding suggests that well-known researchers publishing in high-impact journals are likely to be less affected by citation undercounting than researchers publishing in lower impact journals.

## Is There a Place for OSM?

The possibility of a citation black hole raises the important question of what kinds of material, if any, should actually appear in the OSM. For example, many journals now have an “open data policy” and recommend or require authors to make their raw data available through public repositories (e.g., Dryad, Figshare). These repositories now fulfill some of the roles for which OSM was originally intended (e.g., data accessibility). Furthermore, if information really is essential to understanding the manuscript, such information (and associated references) should go into the actual manuscript itself (or an appendix), rather than being relegated to the OSM. In this regard, space restrictions should become less of an issue for open access and online-only journals. Whatever the future of OSM may be, journals and publishers need to ensure that information currently contained in existing OSM is accessible and that links to those materials remain active.

## Conclusion

Over the last two decades, there has been an increase in the number of citations published in OSM that are invisible to services used for citation counting. These findings pose a global issue and highlight that both individuals and journals are at a potential disadvantage by including citations in OSM. A solution to this problem is urgently required and could include journal indexing of citations in OSM or the inclusion of all citations in the bibliography of the main text, which would require the abolition of maximum bibliography limits.

## Conflict of Interest

None declared.
